# Workplace Mental State Monitoring during VR-Based Training for Offshore Environment

**DOI:** 10.3390/s21144885

**Published:** 2021-07-18

**Authors:** Rumaisa Abu Hasan, Shahida Sulaiman, Nur Nabila Ashykin, Mohd Nasir Abdullah, Yasir Hafeez, Syed Saad Azhar Ali

**Affiliations:** 1 Centre for Intelligent Signal and Imaging Research (CISIR), Electrical and Electronics Engineering Department, Universiti Teknologi PETRONAS, Bandar Seri Iskandar 32610, Malaysia; rumaisa_19000937@utp.edu.my (R.A.H.); shahida_24191@utp.edu.my (S.S.); nur.nabila_25582@utp.edu.my (N.N.A.); yasir.hafeez_g03494@utp.edu.my (Y.H.); 2 Petroliam Nasional Berhad, Kuala Lumpur 50088, Malaysia; mnasir_abdullah@petronas.com

**Keywords:** virtual reality, EEG, mental state, offshore training

## Abstract

Adults are constantly exposed to stressful conditions at their workplace, and this can lead to decreased job performance followed by detrimental clinical health problems. Advancement of sensor technologies has allowed the electroencephalography (EEG) devices to be portable and used in real-time to monitor mental health. However, real-time monitoring is not often practical in workplace environments with complex operations such as kindergarten, firefighting and offshore facilities. Integrating the EEG with virtual reality (VR) that emulates workplace conditions can be a tool to assess and monitor mental health of adults within their working environment. This paper evaluates the mental states induced when performing a stressful task in a VR-based offshore environment. The theta, alpha and beta frequency bands are analysed to assess changes in mental states due to physical discomfort, stress and concentration. During the VR trials, mental states of discomfort and disorientation are observed with the drop of theta activity, whilst the stress induced from the conditional tasks is reflected in the changes of low-alpha and high-beta activities. The deflection of frontal alpha asymmetry from negative to positive direction reflects the learning effects from emotion-focus to problem-solving strategies adopted to accomplish the VR task. This study highlights the need for an integrated VR-EEG system in workplace settings as a tool to monitor and assess mental health of working adults.

## 1. Introduction

Stress response can be seen as a built-in alarm for the body to detect deviations from the balanced condition known as homeostasis, and further activate neural activities to increase arousal and alertness [1]. Although its effects may not be easily visible, prolonged stressful conditions can lead to physical and mental health deterioration. The increase in adults facing high level of work-related stress are observed throughout the world [2,3], more so in the recent COVID-19 pandemic due to perceived stress and financial uncertainties [4]. Aside from the conventional approaches used in psychotherapy for diagnosis and healthcare, researchers are exploring physiological signals of stress responses to help in providing objective evaluations of a person’s mental health. The electroencephalography (EEG) is the most used brain-monitoring method for mental stress because of its portability, high temporal resolution, non-invasive, and low cost advantages [5]. The EEG brain waves (0.5–45 Hz) are strongly associated with different brain responses towards stress and have been used to detect stress [6,7,8].

However, monitoring the EEG signals in an actual workplace setting to assess mental health and stress levels is not feasible as this can disrupt the working routines. With virtual reality (VR), immersive 3D environments based on any real-life scenarios can be developed to provide interactive experience for the users. Rapid advancement in sensor technologies has allowed simpler and cheaper setup that integrates EEG with the VR technology. It has been used in various settings such as healthcare [9,10,11,12], oil production [13,14], workplace [15] and education [16]. With the integration of EEG, researchers have studied various brain functions such as attention [17,18], cognitive load [19,20], motor intention and planning [11], emotional processing [21,22], sense of immersion [23,24], engagement [25] and relaxation [26,27,28]. Although VR has been used with the EEG for meditation and mind therapy purposes, it has not been used as an integrated system to monitor mental health in workplaces.

### Motivation of Study

Workplace stress is unique and depends on the setting and challenges faced by employers within the company. The flexibility of VR to emulate different real-life scenarios makes it an excellent tool to assess mental states and performances of workers, especially in workplaces with high-risk conditions such as offshore facilities. Offshore workers are amongst working adults that face high-level of stress during their working hours [29]. Moreover, the severe impact of COVID-19 pandemic on the offshore industry has changed the standard operating procedures in these facilities [30,31]. The lack of expertise or carelessness in handling the machinery within an oil well facility can lead to accidents and severe injury. Prior to operating at remote locations, workers must undergo extensive training to operate offshore oil and gas production facilities.

Leading oil and gas companies have adopted the VR technology [32] in their training and operations [33]. Conventional training methods for offshore operations involve direct interaction between trainers and trainees where courses are given on how to choose proper tools and operate complex machines under varying conditions. Theoretical and conceptual training such as classroom and e-learning courses lack hands-on training, whereas live-training during actual working hours are costly, high risk and not applicable for dangerous operations. Research has been done to develop VR-based offshore training with different modules such as safety handling [34], crane training [35,36], fire incidents [37,38] and hazard-based emergency response training [39]. Integration of VR technology in the chemical industry has been observed to improve training time and cost [14,40], and time for facility recovery and startup after shutdown [41].

In this paper, we evaluate three different mental states induced when performing a stressful task within a VR offshore environment. We hypothesize that the changes in mental state when performing the VR task of a working environment can reflect specific behaviours which is measurable using EEG. The mental states observed are the discomfort felt due to the VR itself, and changes in stress and concentration levels when performing the task. Findings from this paper contributes to further efforts to integrate VR and EEG to monitor mental health in stressful workplace. The VR offshore environment is developed based on four different training modules, and is assessed on user-experience using questionnaires and performance scores. Section 2 details the developed VR-offshore environment and its four modules, Section 3 describes the testing procedures conducted to assess the VR performance and mental states using EEG device, followed by results and discussion in Section 4 and Section 5.

## 2. VR-Based Training for Offshore Environment

More research is incorporating the VR technology into mental health applications such as in resilience [42], emotion regulation [43], and mindfulness [25,44] interventions. This paper presents the changes of mental states in response to a stressful task performed in a VR-based offshore environment. The VR environment is developed based on four different training scenarios of operating the oil well facilities using the Unity 3D software. Microsoft Visual Studio is used to program the interactions within the environment. The HTC Vive headset is used to display the VR environment and its controllers are enabled to allow hand-movement operations. The HTC Vive is calibrated using Steam VR engine to ensure the efficiency of training during the simulation and provide better immersive-effect in the user. The following section presents the four VR-training modules developed for offshore training.

### 2.1. Virtual Environment for Offshore Training

**Figure 1:**
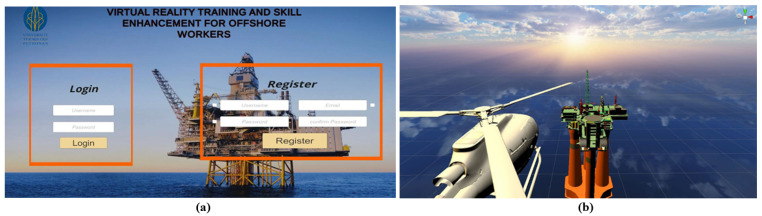
(**a**) Startup interface of the VR-training module and (**b**) the initial scene of arriving at offshore platform.

Figure 1 presents the startup scene of the VR environment. The scene begins with the simulated event of arriving at the offshore platform through a helicopter. Once the user arrives on the platform, the controller can be used to choose and teleport to the preferred training area. Each modules begin with a list of instructions and several tools that will be used during the task. The tools can be chosen and operated using the HTC Vive controllers.

#### 2.1.1. Module 1—Oil Sampling

**Figure 2:**
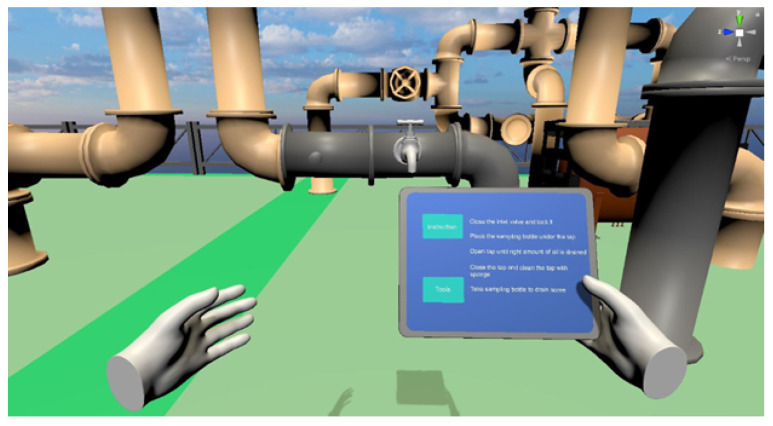
Module 1 Oil sampling.

Oil sampling is an essential routine activity for offshore workers where they are required to collect oil samples for laboratory analysis. Careless sampling or contamination in the sample container can lead to inaccurate results and losses as the whole routine also involves transporting and testing of the sample. Module 1 (as shown in Figure 2) requires the trainee to close the inlet valve and lock it to stop the oil flow into the tank, place a sampling bottle under the tap, open tap until sufficient amount of oil is drained, close tap and clean with sponge and lastly pickup the sampling bottle.

#### 2.1.2. Module 2—Confined Space Inspection

**Figure 3:**
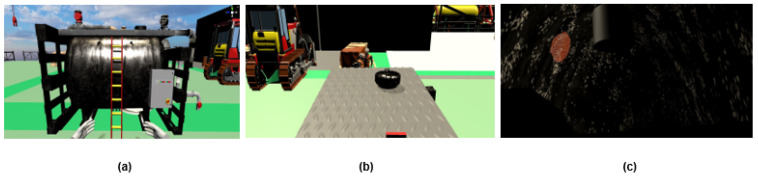
Module 2 Confined space inspection: (**a**) Oil tank with ladder. (**b**) View when standing on top of tank. (**c**) Dark surrounding when inspecting inside of tank.

Working at the offshore facilities often involved performing task within confined spaces such as silos and tanks. This module introduces the trainee to procedures of entering and exiting a tank. Similar to Module 1, trainee must first close the inlet valve to stop the tank process flow. Before climbing the tank, the sensor power board needs to be switched off. For the climbing movement, the trainee must touch the ladder to trigger the climbing animation to the top of tank in the VR environment. The VR environment inside the tank is built to emulate dark surrounding of a small space (as shown in Figure 3). Trainee will enter the tank and inspect for damages inside the tank.

#### 2.1.3. Module 3—Pipeline Valve Rotation

**Figure 4:**
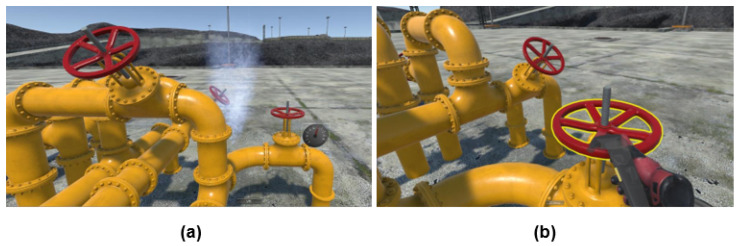
Module 3 Pipeline Valve Rotation: (**a**) Smoke leakage from pipeline. (**b**) Rotating valve using controller.

Managing offshore pipelines is also a crucial operation to ensure safe transportation of oils and gas. In this module, the trainee is required to observe any smoke leakage from the pipeline and rotate the valve in clockwise direction for at least 30 turns using the controller (as shown in Figure 4). The leakage must be contained within a time frame of 35 s, with the speedometer attached to the pipeline is set to reduce at a rate of 2 unit/s to indicate drop of pressure. Scores in Module 3 is based on the number of valve rotation the participant was able to perform within 35 s or until no smoke leakage was observed in the VR environment.

#### 2.1.4. Module 4—Crane Training

**Figure 5:**
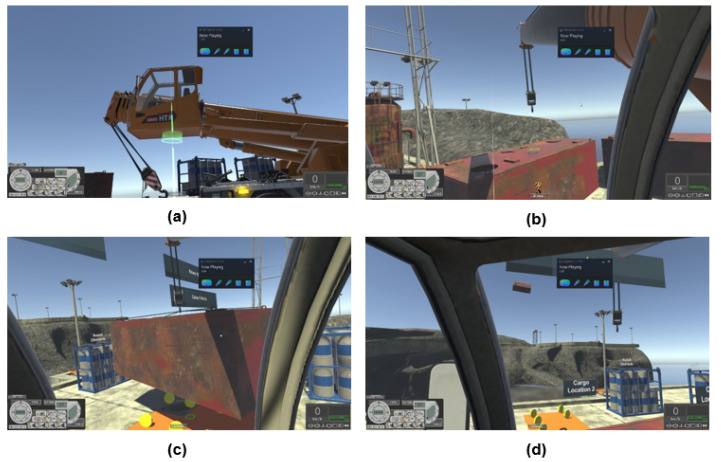
Module 4 Crane training: (**a**) Transporting into crane driving cab. (**b**) Adjusting the lifting hook. (**c**) Lifting cabin whilst avoiding obstacles. (**d**) Example of accident during cabin lifting.

This module allows trainee to experience handling the crane while moving large cabins without any collision. When situated within the crane driving cab, the trainee will control the position of the lifting hook, pick up the cabin using the hook and place it on the allocated area (as shown in Figure 5). There are two cabins that need to be moved. During this module, coin scores are given based on the success of placing the two cabins in the allocated areas (eight coins per area). During the movement of the cabin, any collision with nearby obstacles results in −5% reduction from a 100% score. The number of coins collected indicate proper placement of cabin and the percentage score indicates the task was performed at minimal collision with any nearby obstacles.

## 3. Mental State Monitoring during VR Training

**Figure 6:**
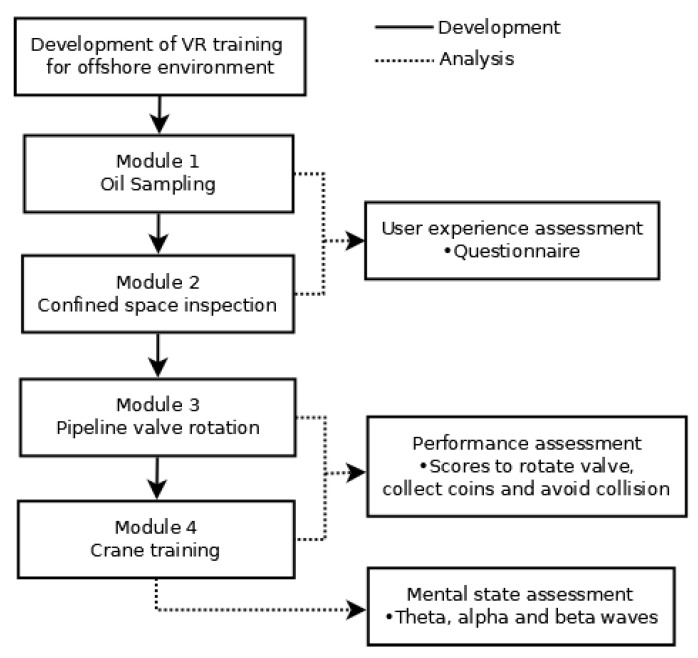
Flow of experiment.

Twenty one participants are recruited to test the developed VR environment for offshore training. Inclusion criteria for the participants are healthy male and female adults, aged 18 or above, and had normal or corrected-to-normal vision. The participants are students from the Universiti Teknologi Petronas (UTP) studying different engineering programs. This study is done under the ethical approval from the Universiti Pendidikan Sultan Idris Ethics Committee (2020-0121-01). Figure 6 presents the flow of experiments done in this study. Module 1 and 2 are used to assess the experience of participants when undergoing the VR training, and Module 3 and 4 are used to assess the task performance of participants. The mental states of participant are monitored using MITSAR-EEG-202-31 device during the Module 4 training.

### 3.1. User Experience and Performance Assessments

In the feedback assessment, participants are asked to choose the most relevant response from Table 1 they experienced after performing the tasks in Module 1 and Module 2. The responses reflect five aspects of user experience from the developed VR environment and can reveal the advantages and drawbacks of the developed VR in emulating offshore operations.

Performance of participants in Module 3 and 4 are evaluated in percentage from the coins collected and ability to avoid obstacles when moving the cabin using crane. There are two cabins with each having a designated placement area with 8 coins floating on the surface. Each coin carry 6.25%, giving 100% score when all 16 coins are collected. For the collision performance, participants began with 100% and receive −5% deduction if they collided with nearby obstacles when moving the cabin using crane.

### 3.2. Mental State Assessment Using EEG

**Figure 7:**
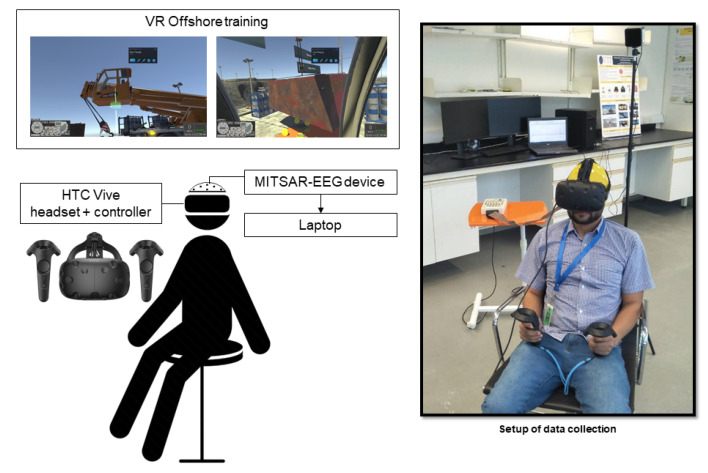
Experimental setup with MITSAR-EEG and HTC Vive headset and controllers.

**Figure 8:**
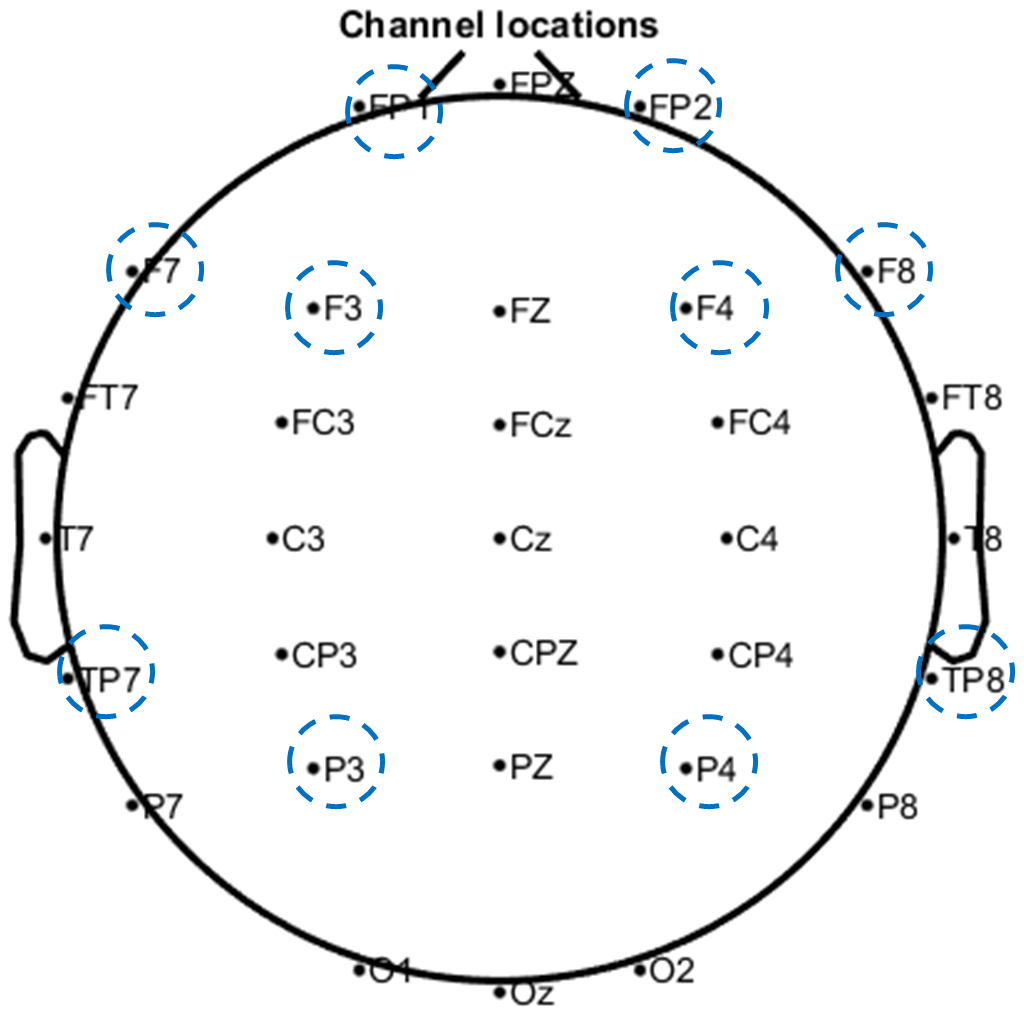
Electrode channels for the MITSAR-EEG-202-31. Circled electrode channels are used for EEG analysis.

Figure 7 illustrates the experimental setup for the EEG recording and the VR headset. The EEG signals are collected using a 32-channel EEG cap with common reference setting and at 500 Hz sampling rate. Impedance for all channels is maintained below 10 kΩ during EEG data recording. Figure 8 illustrates the electrode channels used for this study.

**Figure 9:**
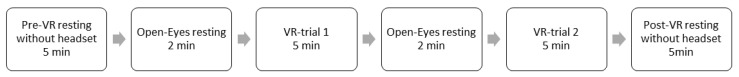
Six segments of the whole EEG data recording.

The whole EEG recording session is divided into six segments as shown in Figure 9. Firstly, the EEG is recorded at resting with eyes-close without wearing the VR headset for five minutes. The participant is then given the VR headset and asked to rest with eyes-open for two minutes. During the eyes-open resting, a black screen is displayed on the VR. The VR trials in this session are given two conditions where the participant must complete the crane training in Module 4 within a 5 min time limit and without colliding with any nearby obstacles. Once the second VR trial is completed, the VR headset is removed and participant is asked to rest with eyes-close for five minutes. The duration of the whole EEG recording is approximately 24 min.

#### EEG Analysis

The EEG signals are re-referenced to Cz and filtered using band pass filter between 4 and 45 Hz. Delta band (0.5–4 Hz) is not analysed as activity below 5 Hz is prone to gross movement artifacts [20]. Independent component analysis is done using the Infomax algorithm (runica, ‘extended’) in the MARA plugin [45] for EEGLAB toolbox [46] to remove motion artefacts, with automatic artefact rejection probability > 95%. Signals are segmented into 30 s epochs to observe the average mental state in each segment (Figure 8).

In this study, only 10 channels were selected for analysis as shown in Table 2. Using the FIR filter, the EEG signals are decomposed into theta (4–8 Hz), alpha (8–13 Hz) and beta (13–30 Hz) bands. Theta band in the temporo-parietal areas has been associated with discomfort and disorientation when using the VR headset [47]. Alpha and beta bands reflect the conscious states of the brain, with increasing stress results in decreased alpha and increased beta activities [48,49]. For the present analysis, both bands are further decomposed into sub-bands: low-alpha (8–10 Hz), high-alpha (10–13 Hz), low-beta (13–18 Hz) and high-beta (18–30 Hz). Each sub-bands reflect different brain responses as shown in Table 1. Relative power for each frequency band is calculated using Equation (1) [50,51].

(1) $$Power_{relative}=\frac{\sum_{i}^{n}Band^{2}}{\sum_{i}^{n}Total^{2}}$$

Within the alpha band, alpha asymmetry (AAS) between the right and left frontal areas is a significant indicator for stress [53,54]. We calculated the AAS between the right (Fp2,F4,F8) and left (Fp1,F3,F7) frontal areas using Equation (2) [54]. Brouwer et al. highlighted the direction of AAS reflects approaches of stress coping [53]. The AAS ranges between [−1, 1], with negative values reflecting higher level of stress in the right frontal areas.

(2) $$AAS=\frac{Right-Left}{Right+Left}$$

## 4. Results

A total of 21 participants were recruited among UTP students for this study. None of the participants had any experience of working on the oil rig platform. Table 3 presents the characteristics of participants recruited for this study.

### 4.1. User Experience Assessment

**Figure 10:**
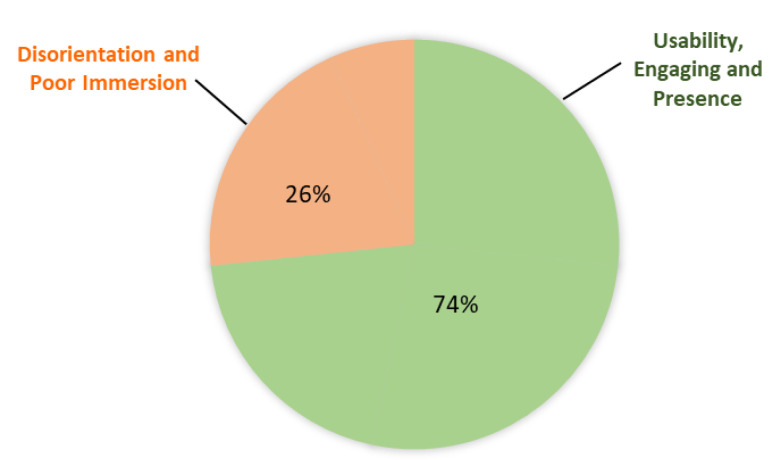
User experience during Module 1 and 2.

Figure 10 presents the percentage of participants based on the most relevant response they experienced when performing the VR modules. As reflected in the green pie slices, 74% gave positive responses on their VR experience with regards to the usability, engagement and sense of presence. The training modules were easy to understand, and motivated participants for future training, with one participant volunteered to perform the training again. The confined space inspection training in Module 2 was able to induce a sense of claustrophobic as participants felt that the training helped them to face their fear of confined space. However, there were participants that felt the VR environment had poor sense of immersion when performing the training. Participants that felt motion sickness during the VR training also informed researchers that they had no prior experience of using the VR headset.

### 4.2. Performance Assessment

**Figure 11:**
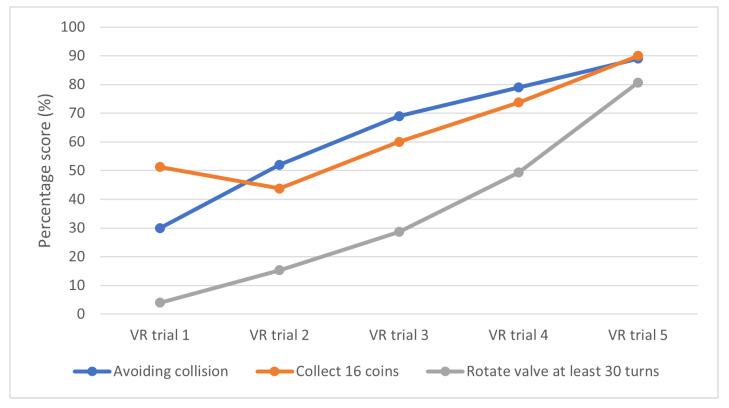
Percentage scores achieved by participants during training in Module 3 and 4 for five sessions.

In Module 3 and 4, the performance of participants improved throughout the five sessions. This is reflected by the increasing performance scores of rotating the pipeline valve and moving cabins using the crane in Figure 11. As more training were given to the participants, they were able to use the VR controller to rotate the valve with more ease, avoid collision with nearby obstacles and place the cabin correctly within the designated placement areas and collecting more coins.

### 4.3. Mental State Assessment

The mental states were assessed for Module 4 where the crane training must be completed within a duration of 5 min by collecting all coins and avoid any collision with nearby obstacles when moving and placing the cabins. These conditions were placed to induce stress in participants as the task must be completed correctly and efficiently. Relative power of the 30 s epochs in each segment is calculated to observe the changes of mental states in the three frequency bands.

#### 4.3.1. Theta Activity

**Figure 12:**
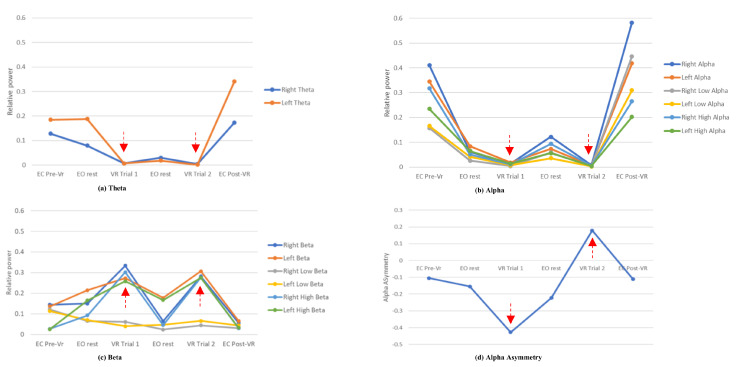
Changes of (**a**) theta, (**b**) alpha and (**c**) beta relative powers, and (**d**) alpha asymmetry throughout the six segments.

Changes in the theta activity are observed in Figure 12a, with decreased relative power of theta during the VR trials. During the second 2 min rest with eyes-open, the theta activity slightly increased but did not reach to the level of theta before VR trial 1. Once the VR trials were completed, the theta activity increased close to the relative power before VR headset is placed on participant. The discomfort and disorientation of VR headset has been negatively associated with relative power of theta [47].

#### 4.3.2. Alpha Activity

With reference to Figure 12, alpha band had the highest relative power in the first and last segments where participant is not wearing the VR headset and in relaxed states. Similar to the theta activity, Figure 12b also shows decreased in alpha activity once the VR headset was worn, and it further decreased during the VR trials. However, unlike the theta activity, both alpha and the sub-bands had comparable relative powers during the eyes-open resting segments. Higher alpha activity indicates a calm and relaxing state, whilst the decrease in this frequency band reflects increased level of stress [48,55]. Both alpha sub-bands had similar changes throughout the six segments suggesting no differences in the level of alertness during relaxed states.

#### 4.3.3. Beta Activity

**Figure 13:**
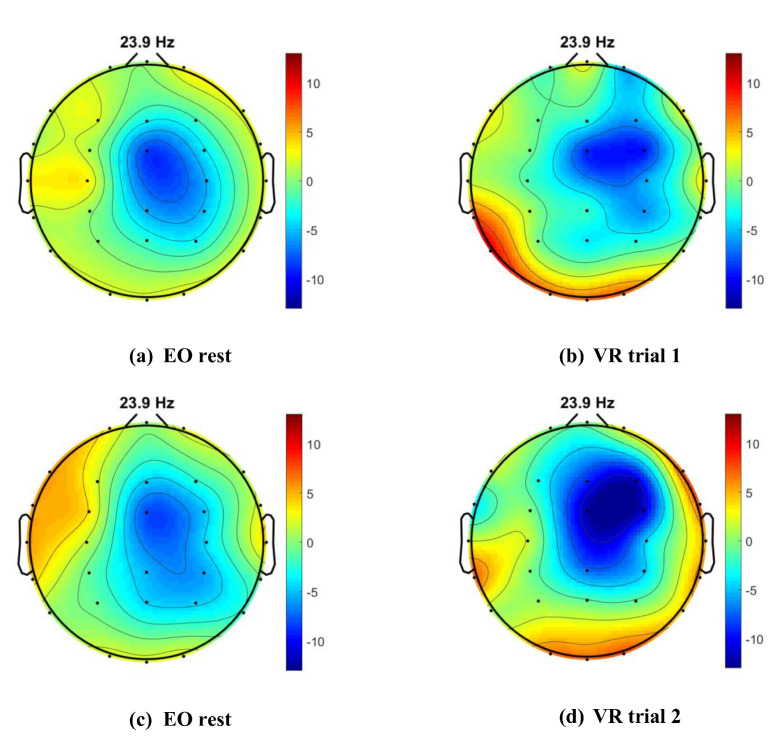
Topomap in high-beta sub-bands during (**a**) eyes-open resting (**b**) VR-trial 1, (**c**) eyes-open resting, and (**d**) VR trial 2. Increase in high-beta activity observed in the posterior areas during VR trials compared to eyes-open resting.

In the beta frequency band, there is an increase of activity during the VR trials as shown in Figure 12c. This is also observed in the high-beta sub-band but not the low-beta sub-band, suggesting increasing mental concentrations but with high emotional intensity during the VR trials [52]. Further analysis of the high-beta sub-band is done using the EEGLAB to extract the topomap of the 32-channel brain activity during resting and VR trials, as shown in Figure 13. The topomap allows us to observe the brain areas that are affected within the high-beta frequency in response to changes in concentration under intense emotions. Increased brain activity is observed in the temporal and parietal brain areas during the VR trials, which is not present during the 2 min resting with eyes-open.

#### 4.3.4. Alpha Asymmetry

The AAS gives an indication of the dominant frontal areas in response to stress, with positive value reflects high alpha activity in the right frontal area. In Figure 12d, AAS values are close to zero during resting for both eyes-open and eyes-close conditions showing similar alpha activity between the right and left frontal areas. However, AAS decreased further in VR trial 1 showing a dominance of left frontal areas, and deflected to positive value in VR trial 2. Direction of the AAS is associated with the strategies taken to cope with stress, with negative asymmetry reflect withdrawal strategies and positive asymmetry for approach strategies [53].

## 5. Discussion

Workplace stress is one of the causes of mental health problems among adults [2,56]. Self-reports of mental health and work-related stress are often under-reported due to concern of societal stigma and backlash of employment [57]. Aside from professional help, conventional approaches of managing mental health usually depend on personal resources or social supports. We propose the integration of VR and EEG system as a tool to manage mental health in stressful workplaces. The VR technology allows the monitoring of mental health of adults in virtual environment of their workplace. This study aims to evaluate the mental states when performing stressful tasks within a VR offshore environment.

### 5.1. Advantages and Drawbacks of VR

The developed VR modules were able to engage users to learn basic operations of offshore facilities. In addition to the positive feedbacks on usability, engagement and sense of presence of the VR modules, the learning effects is reflected by the improvement in the performance scores. The ease-of-use and learning motivation observed from the VR offshore training suggest that this technology is a good platform to train offshore workers on operations within the oil well environments. The scenarios can be programmed to mimic accidents or high-risk situations to train workers, without the need for a real-structure location.

However, these scenarios need to be more immersive and realistic enough to the real-environment. Higher level of immersion and sense of presence have been observed to elicit similar brain activity to the responses in real-life setting [58]. In a VR-based fire extinguishing training, the level of realism during the VR experience improve the interaction and sense of presence in users [38].

With the increase level of realism in a stressful VR environment, precaution must be taken to ensure extreme stress is not induced during the VR experience. Fadeev et al. reported that VR with extreme stress scenarios can elicit autonomic and EEG responses due to high emotional stress [21]. Saghafian et al. also suggested the need to measure realism with emotions within a stressful VR experience [38]. Motion sickness, disorientation and discomfort are also potential stressors when experiencing the VR [47,59]. Measuring these aspects of the VR using questionnaires has the limitation of inaccuracy as the user experiences are reported after the VR session [59]. Using the EEG signals, Liao et al. developed a prediction model to detect motion sickness in VR. The integration of a VR-EEG system that emulates workplace with complex or high risk conditions can ensure a safer VR environment with objective monitoring tools to measure both mental states and user experience.

### 5.2. Presence of Stress

Brain activity in different frequency bands evinces specific brain functionalities [60,61]. Changes in the mental states observed in this study corroborate the presence of stress due to physical discomfort and conditional tasks during the VR trials. Theta frequencies are known as slower brain rhythms and are prominent during unconscious states such as drowsiness [62] and relaxed [63]. Heo et al. observed a negative association between relative theta power in the left temporo-parietal area and discomfort felt during VR gaming [47]. In our study, the decrease level of relative theta power observed between the VR trials suggest disorientation and discomfort when wearing the VR headset [47]. Although these unfavourable spatial effects remained during the 2 min rest between the two VR trials, the increase in relative power of alpha in this segment suggest an increase of relaxed state.

Reduction in alpha activity is a strong indicator of stress as brain activity in this frequency band reflects relaxed state of consciousness [48,61]. Higher relative power in the alpha band compared to theta and beta bands during resting reflects its dominant role during relaxed consciousness [62], and has commonly been used as the neuromarker for stress response state [6,8,23,25]. Change in the alpha activity from high to low indicates increasing alertness and attention to external stimuli, as observed during the VR trials. The deflection of AAS from the zero-baseline also reflects the brain responses in coping with stress [53].

Beta frequency band reflects brain activities with faster oscillation, and has been associated with concentration [64], alertness [65], and attention [66,67,68]. Changes in the beta activity over the sensorimotor cortex has been associated with sensorimotor control and peripheral muscular activity [69]. In our study, the increase of beta activity during the VR trials is dominantly due to the increase of high-beta sub-band (18–30 Hz). This sub-band has been associated with higher level of concentration but with increasing emotional intensity such as anxiety [52] and agitation [68]. Errico et al. used the ratio of high-beta to low-beta sub-bands to calculate alertness index [22] in a study on emotion reactions within the VR. The alertness index correlated positively with distress, and negatively to emphatic interest and empathy. In this study, the findings of beta activity reflects the brain responses involving sensorimotor processes under high level of stress when performing the VR trials. The stress is induced through the VR crane training by placing the conditions of achieving perfect scores within a 5-min duration.

### 5.3. Learning under Stress

Improvement in the performance scores is a marker to show learning occurring throughout the VR sessions. This is also shown from the change of AAS from negative to positive between the two VR trials. According to Brouwer et al., negative AAS indicates withdrawal-coping strategies whilst positive AAS indicates problem-solving strategies when facing stress [53]. The positive AAS observed in VR trial 2 reflects the change in strategies to complete the task successfully. This change is also observed in the reduction of high-beta activity in the topomap for VR trial 2 near the temporal and parietal brain areas. The decrease in high-beta activity shows lesser anxiety felt by participant as compared to the first trial. Both of these brain areas correspond to sensory processing in the brain where parietal lobe is known for spatial and visual perception, and temporal lobe for sequencing and organization [60]. The shift of high-beta activity from left to right posterior area between the VR trial topomaps may reflect improved error detection and complex decision-making during the second VR trial [60].

## 6. Conclusions

In conclusion, this study shows that the changes in mental states due to discomfort, stress and concentration induced when performing VR tasks in an offshore environment can be monitored within the theta, alpha and high-beta frequency bands. The integration of VR and EEG to emulate workplace environments is a viable tool to monitor mental health. In settings where the workers are under high risk conditions or involved in complex routines, the use of EEG in identifying potential stressors can ensure the development of safer and effective VR environments. The mental states monitored when performing tasks in the virtual workplace can also be used to improve learning and performance through VR training modules. The proposed approach of integrating VR-based workplace environments with mental state monitoring can be implemented in various settings such as healthcare, office workplace and high-risk industries to train employees on their working skills.

## Figures and Tables

**Table 1:** How do you feel after experiencing the training modules using the VR?

User Experience		Response
Usability	−	The training is easy to understand
Engaging	−	Excited to try another virtual training
Presence	−	It helps me to overcome my claustrophobia
Disorientation	−	I got motion sickness from the VR headset
Immersive	−	The experience is not immersive enough

**Table 2:** EEG brain activity with respective stress responses.

EEG Wave	Brain Area	Electrode	Stress Response	Sub-Band
Theta [47]	Temporo-parietal	TP7,TP8	Discomfort, disorientation	−
Alpha [52]	Frontal	Fp1,Fp2,F3,	Relaxed and calm	Low
		F4,F7,F8	Relaxed and alert	High
Beta [52]	Parietal	P3,P4	Concentration	Low
			Concentration and anxiety	High

**Table 3:** Characteristics of participants.

Characteristic	N = 21
Age	27 ± 5.90
Gender	9 males, 12 females
Tertiary Education Field	Engineering
Normal or corrected-to-normal vision	Yes
Neurological and physiological complication	No

## Data Availability

The data are not publicly available due to restrictions presented in informed consent. The data presented in this study are available on request from the corresponding author.
